# A Selective Change Driven System for High-Speed Motion Analysis

**DOI:** 10.3390/s16111875

**Published:** 2016-11-08

**Authors:** Jose A. Boluda, Fernando Pardo, Francisco Vegara

**Affiliations:** Departament d’Informàtica, Escola Tècnica Superior d’Enginyeria, Universitat de València, Avd. de la Universidad, s/n, 46100 Burjassot, Spain; Fernando.Pardo@uv.es (F.P.); Francisco.Vegara@uv.es (F.V.)

**Keywords:** CMOS image sensor, event-based vision, high-speed visual acquisition, data-flow architecture, FPGA system, laser scanning

## Abstract

Vision-based sensing algorithms are computationally-demanding tasks due to the large amount of data acquired and processed. Visual sensors deliver much information, even if data are redundant, and do not give any additional information. A Selective Change Driven (SCD) sensing system is based on a sensor that delivers, ordered by the magnitude of its change, only those pixels that have changed most since the last read-out. This allows the information stream to be adjusted to the computation capabilities. Following this strategy, a new SCD processing architecture for high-speed motion analysis, based on processing pixels instead of full frames, has been developed and implemented into a Field Programmable Gate-Array (FPGA). The programmable device controls the data stream, delivering a new object distance calculation for every new pixel. The acquisition, processing and delivery of a new object distance takes just 1.7 μs. Obtaining a similar result using a conventional frame-based camera would require a device working at roughly 500 Kfps, which is far from being practical or even feasible. This system, built with the recently-developed 64 × 64 CMOS SCD sensor, shows the potential of the SCD approach when combined with a hardware processing system.

## 1. Introduction

Most common artificial vision systems are based on full-frame image processing [1]. The representation of a scene in an instant *t* as a still image is the typical source of data to extract visual information. Conventional video systems are based on the sequential acquisition and processing of full-frame images. Independently of whether there have been changes in the scene or not, all of the pixels are acquired, stored and processed, which is not efficient in terms of resources if there are no relevant changes. Moreover, this sequential nature makes it more difficult to reduce the control loop delay in real-time applications.

Nature, with evolution being the key point, has developed one of the most perfect machines any engineer could possibly conceive of: living beings. Engineering has drawn on nature as a source of inspiration to solve many problems, particularly in the field of sensing [2,3]. Biological vision systems do not follow the policy of capturing and sending sequences of full frame images at a fixed rate. The idea of a snapshot sequence is not present in biological systems. Visual systems in living beings are based on different types of photoreceptors, which respond to light stimuli, sending information asynchronously to the upper levels of cognitive systems [4].

A Selective Change Driven (SCD) vision sensor delivers only the pixels that have changed most since the last read-out, ordered by the magnitude of their change [5]. Therefore, an SCD sensor only delivers information that is not redundant; as a consequence, there is an efficient use of time and energy, contrary to conventional vision sensors. Additionally, since this information is ordered and delivered synchronously according to the absolute magnitude of its change, the most significant changes (that are related to higher light intensity variations) will be processed first. This pixel prioritization, based on change ordering, can be relevant for some real-time applications that must be accomplished with time restrictions, because it could not be possible to process all of the events delivered by the sensor. The SCD policy ensures that in the case of time constraints, the most relevant changes will be processed.

### 1.1. Event-Based Sensors

Most sensors that have been inspired by this biological approach are based on the Address Event Representation (AER) model [6,7]. In the AER model, pixels operate as individual processing units and fire themselves according to their spatial or temporal change of illumination level. Moreover, event-based sensors can also be classified taking into account how they transform light into an electrical signal. There are integration-based sensors and continuous conversion-based sensors. Light integration is based on a capacitor that stores a charge, which is proportional to both illumination intensity and integration time. Sensors designed with integration photoreceptors offer better image quality. As a drawback, these sensors lose part of their event-based philosophy, because the integration time degrades the fast event-driven response speed. Instead, continuous conversion-based sensors offer a faster response to the stimuli, better mimicking the visual system of living beings.

Many event-based sensors have been developed up to the present. Several of them are especially significant. For instance, [8] has only eleven transistors per pixel and can work in three event triggering modes: illumination level, spatial contrast and temporal contrast. However, this sensor has a low temporal resolution since it is based on fixed time integration. Similarly, the sensors described in [9,10] have good signal quality, since they are based on integration to a fixed voltage, but with a lower time resolution than the events. Worth mentioning is the so-called Dynamic Vision Sensor (DVS) where each pixel autonomously computes the normalized time derivative from the sensed light and provides an output event with its coordinate when this amount exceeds a programmed contrast. DVS cameras offer contrast coding under wide illumination variation and microsecond latency response [11,12]. Hence, it is possible to track fast motion without special lighting conditions

All of the above-mentioned sensors have their particular advantages in certain circumstances. The recently-developed 64 × 64 SCD sensor [13] presents, in our view, a good trade-off, taking advantage of the data reduction that event-driven sensors have, while keeping a synchronous interface that delivers information when the processing system is capable of processing it. Moreover, the feature of reading-out events ordered by the magnitude of their change contributes to the implementation of systems that can work even without full data processing. The fact of having this computing-oriented interface makes the SCD sensor a good candidate to be easily integrated into an embedded processing system. Additionally, the SCD sensor is the only one that combines illumination level and temporal contrast in continuous time, thus providing high-speed operation in both event and illumination response. The SCD sensor will be described in depth in Section 2.2. There is an earlier SCD sensor, with a 32 × 32 resolution based on an integration photoreceptor, which gave a resolution time of 500 μs [14]. However, the current 64 × 64 SCD sensor takes advantage of a continuous conversion cell, allowing higher working speeds.

### 1.2. Event-Based Systems

The next natural step after event-based sensors is the development of vision systems based on that philosophy. However, a problem arises because there is an inherent contradiction when trying to mimic complex mammal vision systems with a traditional computing system. The human brain is a massive parallel system with nearly 100 billion neurons [15]; its good performance relies on the huge amount of connections and on its parallel functioning ability. However, a traditional computing system is sequential in nature. It is true that this drawback can be partially overcome with parallel architectures, but their performance is still very far away from the vision systems of real living beings.

Neuromorphic systems [16,17] appear as implementations in VLSI (Very Large Scale of Integration) circuits of sensor and neural systems, whose architecture and design are based on neurobiology. These systems try to mimic neuro-biological structures present in the nervous system, and AER fits perfectly into this strategy [18].

There are some examples of neuromorphic systems implemented in full custom chips and some others that use FPGAs as processing elements. In [18], a 32 × 32 convolution chip with a 155-ns event latency and a theoretical throughput of 20 mega events per second is presented. The low latency between the input and output streams in a neuromorphic system they term pseudosimultaneity. In this paper, several experiments with both dynamic and recorded AER stimuli are shown, although the highest speed experiments are performed with simulated data. In [19], another convolution module is presented, with a similar speed performance, but in this case with 64 × 64 pixels. It has been designed to allow many of them to be assembled to build modular and hierarchical Convolutional Neural Networks (ConvNets). Similarly, in [20], a neuromorphic system is implemented mixing the DVS event-driven sensor chip together with event-driven convolution module arrays implemented on FPGAs. Experimental results in this paper are the implementation of Gabor filters and 3D stereo reconstruction systems. More recently, a fully-digital implementation of a spiking convolutional event-driven core that can be implemented in FPGAs [21] has been presented. This system uses a DVS sensor, an FPGA and two USB AER mini boards that send AER spikes through a USB connection to a computer. This system is capable of updating 128 synaptic connections in 12 ns, this being an improvement with respect to previously reported FPGA convolutional event-driven cores.

Some processing systems based on AER sensors show the desired pseudosimultaneity, to reduce the control loop delay to its minimum, achieving in this way real-time performance. Most of them are complex systems with a high quantity of resources, which means they cannot be used in embedded systems. Neuromorphic systems try to implement image processing algorithms mimicking living beings’ neural systems as a guideline. In our view, the asynchronous nature of AER sensors makes the subsequent processing system difficult. Sometimes, the processing stages must deal with an explosion of events that are hard to process. In our opinion, event-based systems tailored to embedded systems should have a traditional synchronous interface rather than a neuromorphic approach, which in the end results in a more resource-heavy, less feasible system.

Many AER systems are usually based on FPGAs, often on several boards, due to the complexity of dealing with asynchronous events. Conversely, our approach, based on SCD vision, uses very few resources. We have developed a high-speed event-based motion tracking system, with just the sensor, a medium-size FPGA and some support components. This characteristic means that the system can be integrated into an autonomous platform or any system with limited resources.

### 1.3. Laser Scanning

High speed object detection and scene mapping is an extensively-investigated topic in computer vision. It is useful, for instance, in autonomous vehicle navigation, where it is important to detect obstacles located in the direction the vehicle is traveling [22]. One of the sensing methods used to measure depth without physical contact is laser-based 3D scanning. It is common for laser-based scanners to generate a huge amount of data, for instance in the case of Laser Imaging Detection and Ranging systems (LiDAR) [23]. There are several solutions to measure distances with laser-based scanners: photogrammetry, interferometry or ToF (Time of Flight). The most common technique is known as active triangulation. This method is relatively easy to implement, giving good results for measuring distances in the range of millimeters to several meters. These scanners mainly consist of a laser line generator and a camera that records the pattern projected onto the surface to be measured. Active triangulation has several error sources that limit its resolution, such as any other measurement technique [24]. Many contributions have been published trying to overcome the drawbacks of this technique. For instance, in [25], some solutions are proposed to increase the accuracy of measurements through sub-pixel resolution. Moreover, the basic configuration sometimes varies in order to minimize the occlusion problem due to laser/receiver distance [26].

Nowadays, additional developments have produced small commercial sensors that are employed in robotic applications. For instance, the SICK LMS 200 sensor (Sick AG, Waldkirch, Germany) is based on the ToF measurement principle, and the Hokuyo URG-04LX scanner (Hokuyo Automatic Co. ltd., Osaka, Japan) uses amplitude modulated laser light. By measuring the phase shift between the emitted light wave and its reflection, it computes the target distance. In [27], there is an in-depth comparison of both sensors. It is shown how, in the case of the Hokuyo sensor, the accuracy is strongly dependent on the target surface properties. ToF techniques have been employed in other commercial scanners, such as the MESA SR4000 scanner sensor (Mesa Imaging AG, Zurich, Switzerland). It has a resolution of 176 × 144 pixels and 50 fps. Similarly, PMD’s CamCube 3.0 (PMD Technologies AG, Siegen, Germany) also uses ToF and achieves 40 fps for a resolution of 200 × 200 pixels or 80 fps for 160 × 120 pixels. An analysis of the state-of-the art in the field of lock-in ToF cameras can be seen in [28]. The performance of ToF range cameras has been improved over the last few years; error sources are minor, and higher resolution and frame rates can be achieved. Despite these improvements, ToF cameras cannot yet achieve the depth accuracy obtained by classical triangulation systems. As a final example, Microsoft’s Kinect is being used nowadays for depth mapping. In [29], there is an in-depth analysis of this sensing system. Similarly, high-speed cameras, devices capable of frame rates in excess of 250 frames per second (typically over 1000), are being used nowadays intensively in many applications [30,31].

The volume of data involved that needs to be processed in real time, together with the power and size restrictions inherent in an embedded system suggest the use of an SCD vision system to address these problems. It has been already proven, with the previous 32 × 32 SCD sensor based on an integration cell, that this approach can be employed to detect the frequency of a rotating movement at very high speeds [32]. In this study, movement detection is done off-line by software in a PC (with the Fourier transform), due to the relatively long delay of the data in the order of tenths of μs. With these experiments, it has been shown that the SCD approach has been able to detect frequencies up to 240 rps, frequencies that cannot be detected with an equivalent classical sensor.

In this paper, we present a real-time high-speed working system, developed with the new 64 × 64 SCD sensor based on a conversion cell. The system is able to track movements in real time. This tracking is done by means of computing an object’s distance. This distance computation is made in real time for each new pixel that arrives, instead of off-line, as reported in previous experiments. As will be shown in Section 3, it has been possible to track a rotating movement with a delay of 1.7 μs. This speed is above one order of magnitude faster than the speed of previous systems. The proposed system can track arbitrary movements in real time, thanks to the use and combination of a new SCD sensor, a new pipelined processing architecture and the use of hardware based on a portable FPGA board. The following sections describe the system in detail.

## 2. System Description

Our original motivation was to build a real-time working system as a proof-of-concept of SCD vision. This sensor combines the advantages of event-based sensors, though it can be used in real embedded systems because of its easily integrated synchronous interface. A good application of SCD sensing is object-distance detection for autonomous vehicle navigation. With a few resources, the proposed system should be able to detect objects moving at very high speeds, something that a conventional vision system would not be capable of. Relative movement between the camera and the detected object is necessary. Either the vehicle with the detection system or the obstacle if the vehicle is stationary must be moving to generate a stream of changes. This condition could appear to be a serious restriction, but in fact, it is not. If the vehicle is moving, objects in the moving direction can be detected. If the vehicle is stationary, approaching objects can be detected. Only if there is no relative movement between the vehicle and the obstacle would the object not be detected. In this case, this would not cause any problems.

**Figure 1:**
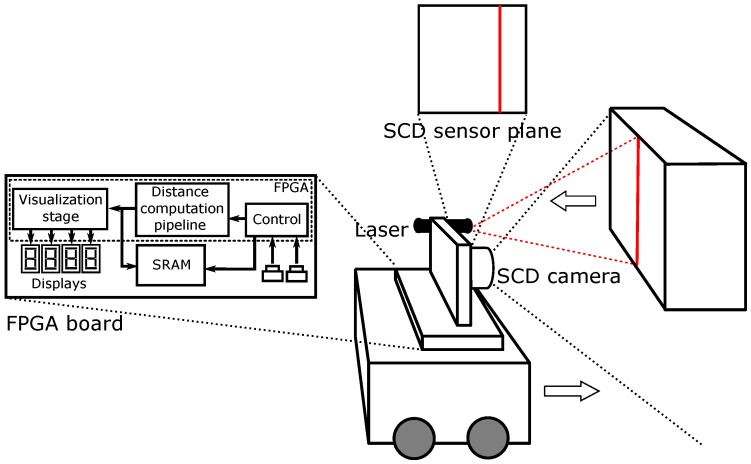
Motion detection system.

Figure 1 shows the system conception. In order to push the system to the limit instead of a real, slow moving vehicle, we have tested the system with the fastest mechanical system available in our lab: a high-speed rotating tool.

The system, as Figure 1 shows, is based on active laser triangulation. The detection system must be set in front of the moving vehicle. There is a laser that projects a line in front of the camera. In the case of Figure 1, the laser is projected onto a moving object. The laser line is captured by the SCD camera, placed at a known distance from the laser. The position of the laser in the sensor image gives the distance between the camera and the surface, as explained in Section 2.1. In fact, for a fixed *y* position (row), the *x* column will give the horizontal distance to the obstacle. As long as the vehicle moves, the laser image will change when there are distance variations. In any case, the SCD sensor will only deliver pixels that have changed. This characteristic dramatically reduces the amount of data to be processed. Of course, it is possible for each row to obtain a different column value. The line can be projected onto an irregular surface, so the line image will give a different column for each different distance, providing an exact depth map. This task is not complicated because as there are only 64 columns and each column position is bi-univocally related to a distance, then it is possible to implement a Look-Up Table (LUT) to instantaneously calculate each pixel distance. This distance profile can be sent to the vehicle control, which decides what to do. Instead of a LUT, because we have assumed that the object has a regular surface, we have decided to compute the average distance of the surface profile. This calculation is an example of how a system can take advantage of the SCD architecture. Each new pixel arrival updates its contribution to the average distance computation, being obtained a new distance value with each pixel. The same algorithm using a classical approach would have required a complete image acquisition. Afterwards, it would have been necessary to binarize the image taking into account only the pixels illuminated by the laser. Then, the average column value of these pixels would have been employed to compute the distance. In our system, each new pixel produces a new distance value in the fastest possible way.

### 2.1. Laser Triangulation System

**Figure 2:**
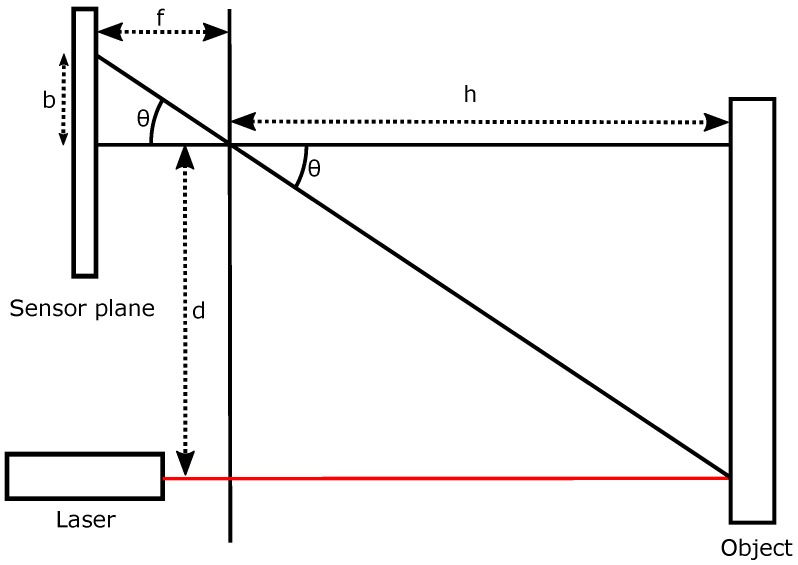
Simple pin-hole triangulation scheme.

As already mentioned in Section 1.3, active triangulation is an easy, widely-used technique for non-contact distance measurement. There are some differences in the method implementation, which depends on the final goal; accuracy, range, etc. Some well-known configurations can be seen in [29] or in [33]. Equations in those papers give accurate values when all of the system variables are known. After all, if extremely high accuracy is not a key factor in the experiment, as in the case of our SCD proof-of-concept, it is very common to use simplified formulae. Figure 2 depicts a simplified pin-hole representation of triangle equivalences shown in those papers. The basic principle of the method consists of projecting a pattern of light (usually a laser line) on the surface to be measured. Afterwards, the pattern image is captured in the sensor plane. Because the laser line is so narrow and the sensor has only 64 columns, it is almost guaranteed that in the range of operation, the laser line will be in one column, or two, while it changes from one column to the neighboring column.

From Figure 2, it is possible to infer:

(1) $$h=\frac{d}{tan(\theta )}$$

Equation (1) gives the value of the distance *h* between the laser-camera system and the surface to be measured. It can be obtained from *d*, the known laser-camera gap, and the angle *θ*. This angle can be obtained as a function of the shift in the image plane (in pixels).

In a linear model, the angle *θ* can be expressed as:

(2) θ=x·ω+ϕ

where *x* is the distance in pixels from the computed pixel to the image center, *ω* represents the radians per pixel and *ϕ* is a useful parameter for alignment error compensation. Hence, Equation (1) can be rewritten as:

(3) $$h=\frac{d}{\tan(x\cdot \omega +\phi )}$$

It is possible to easily obtain *x* from the sensor data stream (in fact, it is the column value), but *ω* and *ϕ* must be obtained through the calibration process; this is shown in Section 3.1. The system range is adjusted by computing these parameters. Of course, higher polynomial fitting would give more accurate values of the angle *θ* as a function of column *y*. This higher precision has a major drawback: higher Programmable Logic Device (PLD) complexity and, consequently, a higher delay, since all of the computations must be made by hardware in the FPGA. Nevertheless, a coefficient of determination $R^{2}$ of 0.9984 has been achieved with the linear adjustment, as is shown in Section 3.1. This coefficient of determination corresponds to a percent of standard deviation of 96%.

### 2.2. SCD Sensor

The first version of an SCD sensor has been designed [34]. Although it is just a 32 × 32 sensor based on an integration cell, it has shown its utility in resource-limited systems [35]. Recently, a 64 × 64 SCD sensor has been designed based on a conversion cell, which will allow it to take advantage of higher working speeds [13].

**Figure 3:**
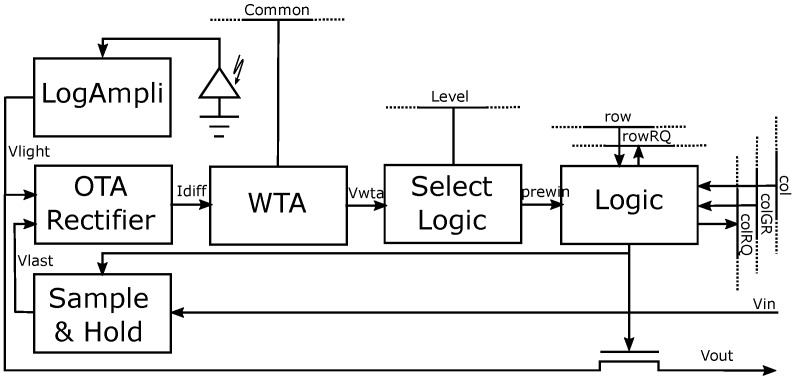
Basic SCD cell.

The basic SCD sensor cell scheme can be seen in Figure 3. This sensor has an array of 64 × 64 pixels. Each pixel can detect whether it has experienced the largest change in illumination since the last time it has been read-out. Any pixel can detect if it is the winner because there is a Winner-Take-All (WTA) circuit that decides which is the pixel with the greatest change. The winner selection has two stages: the first one consists of an analog WTA that selects the set of pixels that have changed most. This set usually already has one single pixel, but in the case of several potential pixels, the second stage digitally selects one of them. All this winner selection takes place in less than 1 μs.

A photodiode transforms incident light into current in each cell. This current generates a logarithmic dependent voltage with a dependency through a log-amplifier configuration, based on a weak inversion transistor negative feedback amplifier and a source follower. The signal *Vlight* is the logarithmic voltage of the incident light. There is a sample and hold circuit, which stores the last read-out value (*Vlast*) in a capacitor. All pixels compare the difference between their last read-out value *Vlast* and the present incident light *Vlight*. This absolute difference is calculated using an Operational Transconductance Amplifier and rectifier (OTA rectifier) that transforms it into the current *Idiff*. All of the *Idiff* currents are compared through the WTA circuit. The *Common* line, shared by all WTA circuits in the sensor array, allows any pixel to generate the *Vwta* signal. The WTA is designed to pull-down the *Vwta* of the winner pixel and pull-up the *Vwta* of the rest. Because there are 64 × 64 = 4096 competitors, it is possible to have more than one pixel signaled as a winner. All of these pixels have their *prewin* signal asserted, and all of them will enter a second-stage competition to select just a single winner. The logic block allows only one of the columns of the attempting winners, setting the *colGR* of the selected column. Each pixel detects this *colGR* setting its row request *rowRQ*, because again, there could be more than one pre-winner pixel cell in the selected column. Immediately, an arbitration circuit decides a single row winner, giving a final winner pixel. This winner will not be sent out until the sensor receives an external clock signal. This signal latches the column and row winners, so the winner will set its *win* signal when both *col* and *row* are set. The sample and hold circuit, triggered by the *win* signal, charges the capacitor to *Vlight*. Consequently, since there is now no difference, the pixel loses the present competition and un-sets the *prewin* signal. The *col* and *row* signals remain unchanged until a new clock signal is received.

**Figure 4:**
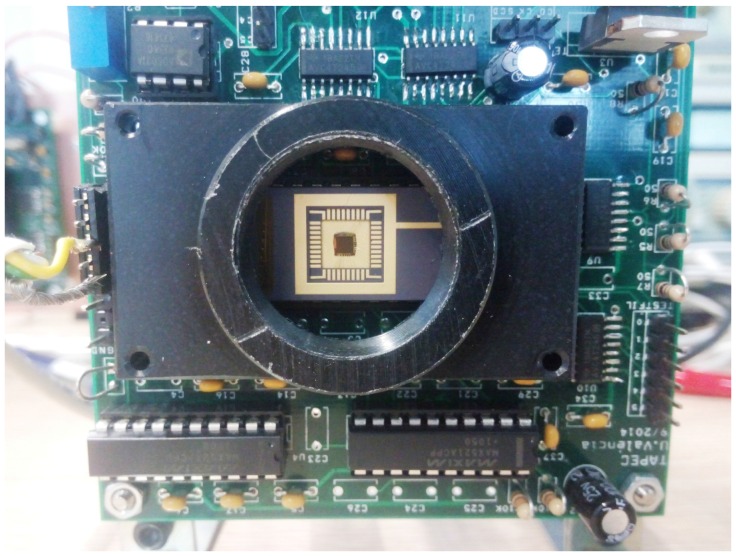
Details of the SCD sensor in the camera.

The chip, which can be seen in Figure 4, has been designed with 0.18 μm 6M1P (6-Metal 1-Polysilicon) MIM (Metal-Insulator-Metal) CMOS technology. A single pixel uses 41 transistors and occupies 30 ×30 μm${}^{2}$ with a fill factor of 4%. Additionally, the sensor has the feature of working as a conventional camera. The sensor has an input signal, *SCDena*, which selects whether the camera works following the SCD function (if set to one) or whether it works as a conventional camera. In the latter, the pixel address must be supplied in order to obtain the corresponding illumination value as a random access memory. This characteristic is useful for system calibration, as is shown in Section 3.1.

### 2.3. SCD Camera

One of the main advantages of the SCD sensor, compared to other event-based sensors, is that it offers a simple interface. The sensor always works as the slave of a processing unit to which it communicates in a synchronous way. A SAM4S Xplained pro micro-board (Atmel Corporation, San Jose, CA, USA) has been used to implement the SCD camera. The camera offers a USB interface to a computer and digitalizes the analogue illumination level value obtained by the sensor. Both functions have not been used in the system, since the pixel stream control has been carried out directly in the FPGA, and the illumination value is not being used. Nevertheless, the camera has been kept in the system because the sensor needs nine polarization analogue values. The camera also adapts the voltage levels between the sensor (1.8 V) and the FPGA (3.3 V). Figure 4 shows the sensor and part of the camera. Similar to most AER systems, only the event address has been taken into account. This has been done in this way in order to reduce the 2-μs conversion time needed for the analogue to digital conversion.

**Figure 5:**
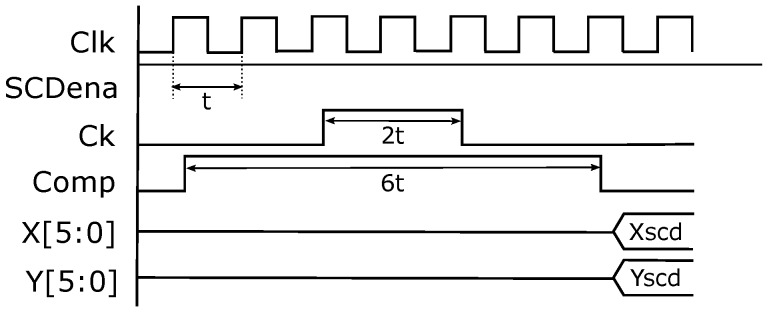
Sensor protocol and timing.

The sensor always sends the pixel that has changed the most based on an external request. Figure 5 shows the timing schema for this. Initially, the competition signal *Comp* must be asserted. After that, the *Ck* signal must be set to one and then set to zero again to generate a pulse while *Comp* is asserted. Then, *Comp* can be released. A few nanoseconds later, the column and row of the pixel appear in the sensor bus. Exact signal timing has been tested with FPGA system clock multiples (t=20 ns period), the event generation being stable with the timing shown in Figure 5. A Finite State Machine (FSM) in the FPGA is in charge of generating these signals. As a conclusion, it is possible to generate a new event each 120 ns in our system, which would be the highest temporal resolution of the sensor without the illumination level.

### 2.4. High-Speed Computation Pipeline

Our assumption is that due to laser brightness, the most significant changes will mostly occur in the laser line. Equation (3) gives the distance of the point where the laser is being projected depending on *x*, the column position of the laser line in the sensor. In our demonstration, we have assumed that there is an almost constant distance where the laser line is being projected. Of course, different values of *h* could be obtained for each different *x*. In this case, and because there are only 64 possible *y* values, a simple LUT with the 64 possible pre-computed depth values would solve the problem. However, our system is not just a pre-computed LUT. Ideally, all of the lines will have the same column, but in a real situation, there would be different values for the columns corresponding to the 64 rows. Therefore, our system computes the average column value, or *x*, as an average distance from the object.

**Figure 6:**
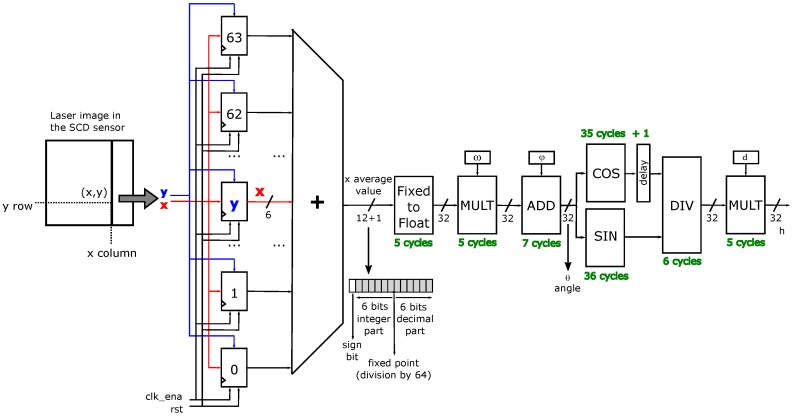
Computation stage.

Figure 6 shows a scheme of the computation pipeline. There is a column of 64 registers, one for each row. When an event arrives with its (x,y) coordinates, the *y*-th row updates its column value *x*. Each row in the registers column stores the present laser position in the image, and it is updated as soon as there is a change. All column registers are added with a tree of carry-lookahead adders. Due to the reduced number of bits involved, these kinds of adders are usually fast enough. The limitation would appear if the adder delay exceeded the clock period; taking into account that the FPGA clock has a period of 20 ns, simulations proved that this restriction is not going to be exceeded. As will be shown later in Section 2.6, the maximum system clock after synthesis is 97.84 MHz, which means that the delays in the carry chain of the adders are below 10.3 ns. This is the expected result since these adders have 12 bits [36].

The maximum result of the addition of 64 registers of six bits fits in a 12-bit register, but an additional bit has been added to the left with a zero value. This has been done to guarantee that the result is interpreted as positive in the next stage. The average value must be computed by dividing the sum by 64, something that is easily done just by moving the point six bits to the left. This operation converts a natural number into a real fixed point number, as Figure 6 shows.

Once the average column value has been computed, Equation (3) must be applied in order to obtain the depth related to that column displacement. To do so, the Altera Library of Parameterized Modules (LPM) has been used since the FPGA employed is the Cyclone II 2C35 from Altera (San Jose, CA, USA). The sequence of mathematical operations that can be inferred from Equation (3) will be done by hardware, as Figure 6 shows.

First of all, the average column value must be converted from fixed-point representation to IEEE 754 floating-point representation, in order to serve as input to the subsequent modules. Secondly, the *θ* angle is calculated by first multiplying *x* by *ω* and then adding the *ϕ* parameter. Next, the cosine and sine of this angle are computed. Because the cosine calculus is one cycle shorter than the sine, a one cycle delay is necessary to equalize the delay in both paths. Afterwards, the division of both magnitudes is performed in the subsequent LPM module, this result being finally multiplied by *d*.

The latency of each LPM module can be seen in Figure 6. Adding these values, the latency of the system is 64 cycles. This is not a bad result, taking into account the complexity of the operations performed. The system has been successfully compiled in the target FPGA obtaining a clock frequency of nearly 100 MHz, which gives a latency of 0.6 μs. Nevertheless, it has been decided to utilize the 50-MHz system clock generated by the board, so the initial latency is 1.3 μs. After this, the system can give a new result each clock cycle, that is each 20 ns or even each 12.5 ns with a 100-MHz clock. In any case, the bottleneck of the system is not the computation pipeline, but the sensor data stream. An FSM needs six cycles to obtain each new pixel event, as shown in Figure 5.

All of the LPM modules have been slightly tuned to reduce their bus widths and the number of pipeline stages. Moreover, the synthesis parameters have been fine-tuned towards maximizing speed instead of reducing area.

### 2.5. Display Stage and Memory Access

Our system is focused on taking advantage, through specialized hardware, of the high-speed resolution time achieved with the SCD sensor. This system is beyond any other previous system using an SCD sensor. Once the distance to the moving object has been computed, it can be used to activate some actuators or just help to make decisions in an autonomous navigation vehicle. In our case, we implemented a simple display system to see, in real time, the computed distance. This has been useful to gauge the accuracy of the system at low speeds. Nevertheless, in order to prove the correctness of the computed data in high-speed experiments, the implemented control FSM stores distance data in an external SRAM (Static Random Access Memory), which is in the FPGA board. Afterwards, data can be extracted from the SRAM and plotted.

**Figure 7:**
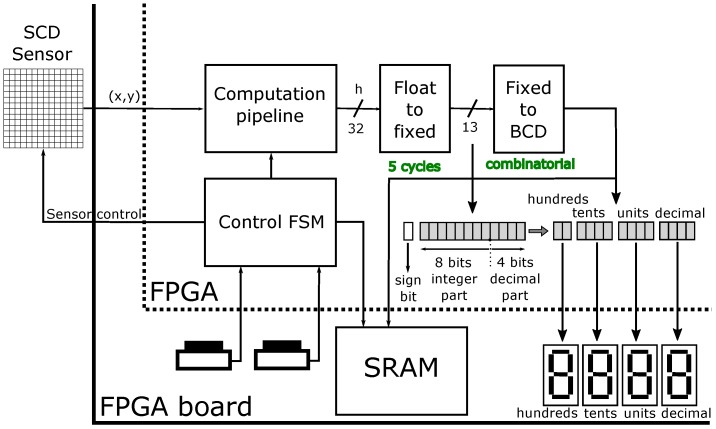
Display and memory stages.

Figure 7 shows a schema of the system with the display stage, the SRAM and the control FSM. In order to visualize distance data in real time, distance is converted to a fixed point representation using an LPM module and then to BCD (Binary-coded decimal) and seven-segment format through a custom combinatorial module. The involved magnitudes are estimated to be from millimeters to roughly two hundred centimeters. The distance between the laser and the camera *d* has been expressed in centimeters, so *h* will also be expressed in centimeters. This has been done with the intention of representing these figures in the display. The theoretical range of distances achieved with the triangulation system would be, as later shown in Section 3.1, in the order of 250 cm. To express these figures, eight bits are necessary for the integer part. Consequently, the decimals appearing as a result will be millimeters. Unfortunately, the low sensor resolution makes it difficult to detect the laser when it is in the range of more than roughly 100 cm.

### 2.6. Synthesis Details

The FPGA board used is the Altera DE2 board. This board contains a Cyclone II FPGA device, with:

- 33,216 logic elements
- 105 M4K RAM (Random Access Memory) blocks
- 483,840 total RAM bits
- 35 embedded multipliers
- 4 PLLs (Phase-Locked Loops)
- 475 user I/O pins
- FineLine BGA (Ball Grid Array) 672-pin package.

The synthesis results shown in Table 1 justify the use of this device. There is still room to add some improvements, although all of the multipliers have already been used.

The FPGA board has many interfaces and additional hardware, although only a few buttons, four seven-segment displays and the SRAM have been used to store the results in the experimentation process. The SRAM is a 512-Kbytes chip, organized as a 256 K × 16, with a 10-ns access time.

## 3. Experimentation

Most smart sensors are developed in CMOS technology because it is possible to include some processing or “smart” capabilities added to the sensing part. Unfortunately, CMOS technology offers worse image quality compared to older CCD technologies. CMOS vision sensors have some noise problems that can be overcome with some additional strategies. In the case of our system, the illumination level has been discarded, and only the events have been taken into account.

There is a source of noise that must be faced in most event-based sensors, the Random Telegraph Signal (RTS). This effect causes some pixels (hotspots) to behave randomly, registering large changes when they should not and, consequently, firing wrong events [37]. This source of noise is related to the presence of traps in the transistor channel and affects a really low quantity of photosensors. The practical effect of RTS noise in a CMOS imager is that it invalidates a few pixels. Each sensor must be characterized to find out which pixels must be marked as faulty. In the case of the 64 × 64 SCD sensor used in our system, only 28 pixels were discarded, giving a ratio of 0.7%. The control FSM incorporates a module that identifies the received pixel, and it is not sent to the pipeline if it is in the faulty pixel list.

The experiment was focused on proving the high-speed capabilities of the system, but beforehand, some low-speed experiments were performed in order to characterize the sensor.

### 3.1. Calibration

Equation (1) expresses the relationship between the object distance *h* and the angle *θ*. In Equation (2), a linear relationship between the angle variation and the column value has been supposed. Finally, Equation (3) gives the object distance as a function of the column number *x*. Since the SCD sensor can work in conventional mode, it has been possible to obtain the column number *x* of the laser in the image plane for several *h* values. The value of *d* has been fixed to 7.5 cm, and the angle θ=arctan(d/h) has been represented.

**Figure 8:**
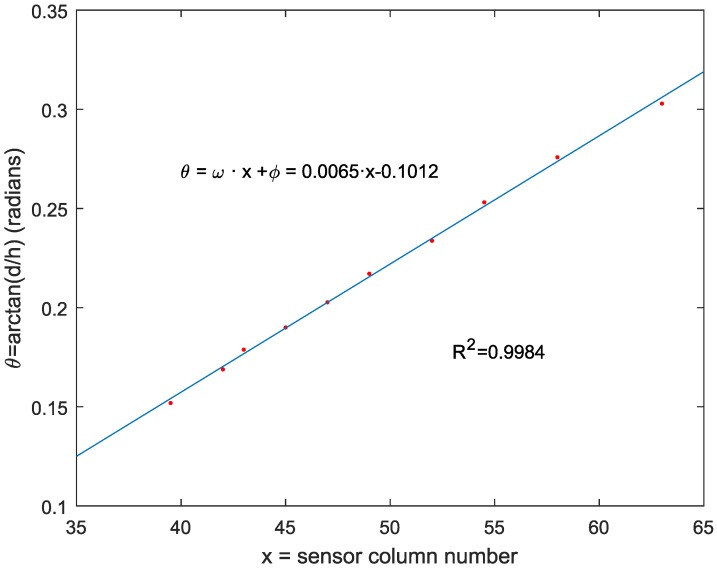
Least squares adjustment of sensor parameters.

Figure 8 shows the least squares adjustment of theta as a function of *x*. This adjustment gives a coefficient of determination $R^{2}=0.9984$, which justifies, in our view, the linear model for *θ*. The linear equation obtained was θ=ω·x+ϕ=0.0065·x-0.1012. These values give a theoretical distance range between 23 cm, as the nearest detectable point, and roughly 250 cm as the farthest measurable distance. Unfortunately, due to the sensor resolution, it has not been possible to keep this range of distances with a single lens. The system has been proven to work effectively in a range up to roughly 100 cm.

**Figure 9:**
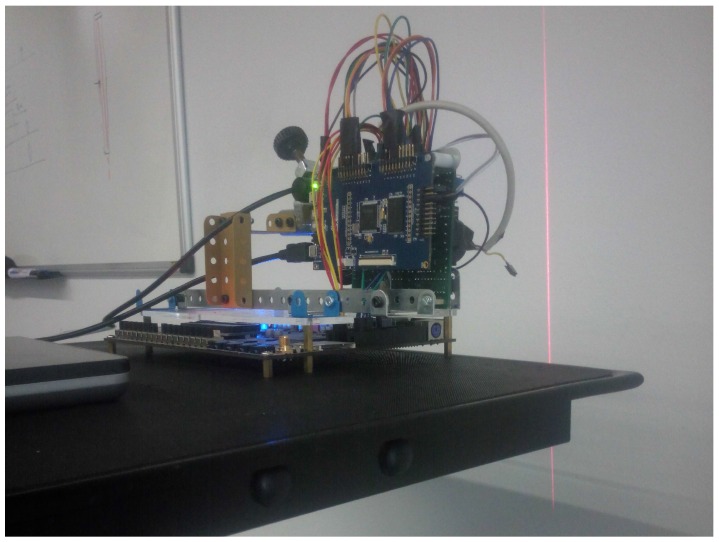
System on the upper part of a wheeled cart in the approaching wall experiment.

Three supply sources are needed for the system: one 9-V supply for the FPGA board, a USB 5-V connection to power the camera and another 5 V to power the laser, which has also been taken from a USB connection. A portable battery with these supply sources has been employed, making a fully-portable system. Everything was set on the top of a wheeled cart, moving it in a controlled environment. Figure 9 shows the upper part of the system and the laser projected onto a wall. With the sensor parameters already computed, some low-speed controlled experiments were performed to verify that the system was working correctly.

During start up, the system may give wrong distance values. This happens because initially, all of the registers do not store the right column values. This is going to happen until all of the registers that store the row value for each column are updated, as Figure 6 shows. It is necessary to update the row of 64 pixels where the laser is projected, but this happens very quickly because these changing events are going to be the first events to be sent out and processed. This is guaranteed by the SCD policy of first sending the pixels that have changed most.

**Figure 10:**
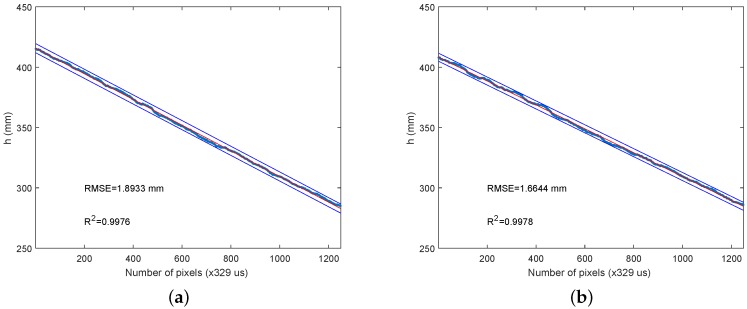
(**a**) Approaching a white plaster wall experiment; (**b**) approaching a blue painted wooden door experiment.

Several low-speed experiments, with different surfaces, have been carried out to test the accuracy of the system at low speed. All of the experiments have been repeated until two standard deviations in confidence have been obtained. The first experiment has been to move the wheeled cart towards a white plaster wall to measure the distance. The experiment needed to be repeated five times to achieve the required degree of certainty. Figure 10a shows the results of the average distance versus time (a new event each 328 μs), with a Root Mean Square Error (RMSE) of 2 mm.

**Figure 11:**
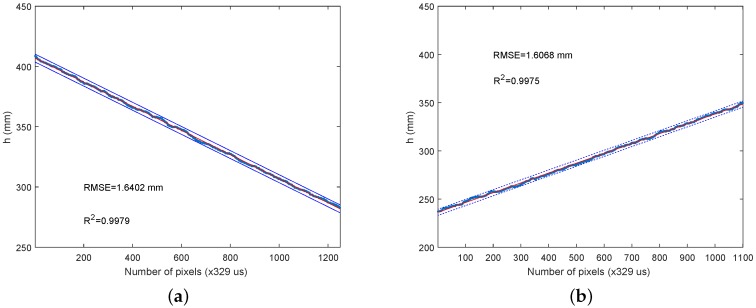
(**a**) Approaching a brown paperboard experiment; (**b**) moving away from a brown paperboard experiment.

The second experiment has been carried out with a blue painted wooden door. Figure 10b shows a better result in terms of less error and a better coefficient of determination. In fact, this second experiment has been repeated only three times, since the required level of certainty has been achieved with three measurements. Additionally, a third experiment with a brown paperboard sheet fixed to a wall has been performed, as Figure 11a shows, with slightly better results.

Better results could be expected with the white surface, since that color is clearer, and it offers greater laser reflection; but this expectation has been proven to be wrong. The brighter color has been shown to be worse for laser line definition in the sensor plane. Nevertheless, experiments done with flatter surfaces and matte colors have worked better. Figure 11b shows the inverse experiment of moving the wheeled cart away from the wall with the brown paperboard; this has been repeated three times.

### 3.2. High-Speed Experiments

The system challenge is to test its movement detection capabilities at high speed. This is really one of the most important facets of any event-based vision system. The delay loop in a control system can be reduced to the minimum possible, because only pixels that have changed are acquired and processed. In our view, the SCD system has the additional advantage that it offers a constant event rate controlled by the processing system, and it also has a very simple interface. This fact allows very high-speed movements in an embedded system to be analyzed, with very few resources. To do so, the fastest tool that we could find was used: a rotating tool with a theoretical maximum speed of 33,000 rpm with no load.

**Figure 12:**
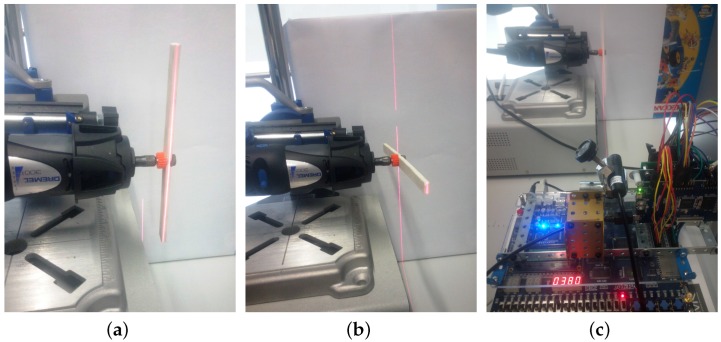
Rotating stick experiment. (**a**) Stick side fully illuminated by the laser (minimum distance to the camera); (**b**) Background mostly illuminated by the laser (maximum distance to the camera); (**c**) Overall experiment set-up view with the rotating stick.

Figure 12 shows three pictures of the experimental setup. A plastic stick is fixed to the rotating tool, with the laser beam illuminating the stick in a perpendicular manner. When the stick is fully vertical (Figure 12a), it offers the maximum surface to the laser beam, being fully detected and giving the minimum distance to the system. Conversely, when the stick is fully horizontal (Figure 12b), it offers the minimum surface to the laser beam contributing, in this case, mostly the background to the average distance.

**Figure 13:**
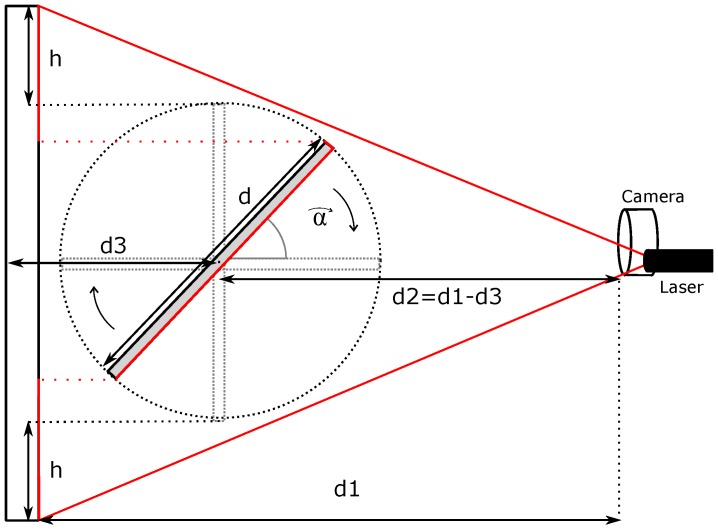
Parametrized model of the rotating stick experiment.

The display is not going to be useful in this case, because the figures change so quickly. Instead, the recorded file in the SRAM is going to be especially useful. Moreover, a theoretical model of what is going to be measured as the average distance in the experiment has been developed. Figure 13 shows a parametrized scheme of the experimental setup.

The stick is rotating at a constant speed of *α* radians per second. There are some assumptions in the model to simplify it: only the laser projection is considered to generate a change in the image, and the laser beam fully occupies one column and only one column. The stick width and the rotating tool axis are not taken into consideration. The upper and lower part of the image are fixed in a symmetrical scenario. The image column received, that is the lateral view of Figure 13, is discretized into 64 rows, so the model adds the contribution of each row and computes the average value, exactly as the proposed system does.

**Figure 14:**
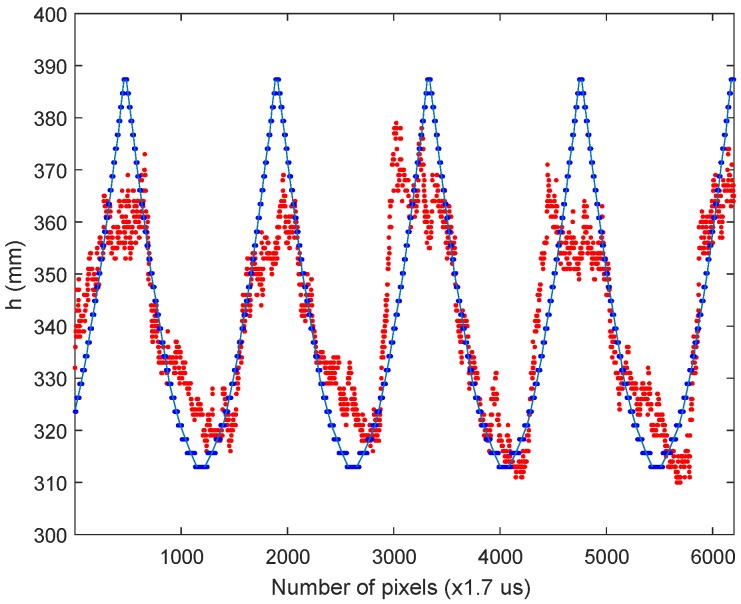
Rotating stick at 12,400 rpm. Red points: experimental data. Blue line: predicted data by the model.

The first experiment performed was done at the lowest speed of the rotating tool. Figure 14 shows the distance position obtained experimentally (red points). In this experiment, an event has been received each 1.7 μs. Experimental data show that the tool is rotating at roughly 12,400 rpm. There is a high coincidence between the theoretical model (blue line) and the experimental result. The coincidence is not absolute, because the model is quite limited, but the periodic movement is perfectly detected. The magnitude of the involved distances is quite coincident, as well.

The experiment shows that a pixel can be acquired and processed each 1.7 μs. A frame rate of 588 Kfps would be needed to achieve this temporal resolution with a conventional camera. A frame-based camera working at that speed is almost impractical, apart from the extremely high required data bandwidth or processing power. It is true that in a conventional system, we would obtain full frames each acquisition time, but there is no way of acquiring, storing and processing such a rate of frames in an embedded system. The SCD system has been able to track a very high-speed movement with very low resources. An equivalent traditional system is not capable of tracking the movement of an object with so much detail at such a speed.

**Figure 15:**
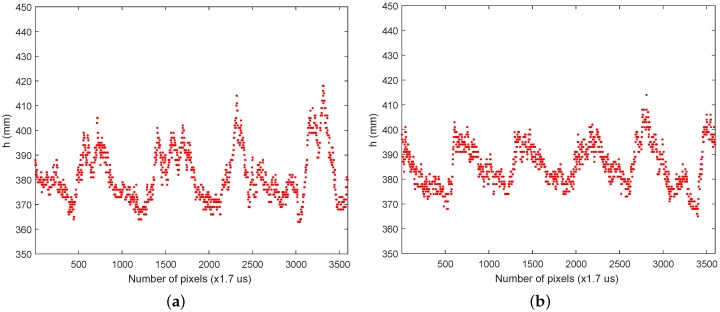
(**a**) Rotating stick at 21,000 rpm; (**b**) rotating stick at 26,000 rpm.

More experiments were performed with the rotating tool, changing some parameters and, more importantly, increasing the tool speed. Figure 15a shows the experimental results with the tool rotating at 21,000 rpm. Again, the periodic movement can be reconstructed, and there is some correspondence with the theoretical mode; for instance, some of the wave peaks are quite sharp as predicted by the model. At this rotation speed, some uncontrolled vibrations appear, which makes it difficult for the laser to point to the side of the stick.

Finally, Figure 15b shows the stick distance at the maximum speed achieved with the rotating tool. This speed is roughly 26,000 rpm, less than the theoretical speed of 33,000 reported in the tool data sheet. We suppose that the tool’s maximum speed must be without any load or just with the tool drills. The stick’s inertia and air resistance are probably why the theoretical maximum speed could not be achieved. In this experiment, the periodicity of the movement is also perfectly detected, and the sharp form of the curves corresponds to the theoretical model. Again, vibrations appear in the system, which make it difficult to obtain more accurate results.

## 4. Conclusions

The combination of SCD sensing, which is a case of event-based sensing, together with full custom hardware processing gives a temporal resolution of 1.7 μs, which is about three orders of magnitude better than that offered by frame-based systems, with even fewer processing requirements. With this temporal resolution, it is possible to track movement at very high speeds, which would be almost impossible with a classical full frame approach. Theoretically, it would be necessary to have a camera working at more than 500,000 fps in order to achieve a similar temporal resolution. Moreover, the hardware needed to acquire, store and process such a massive data stream would be beyond any practical application and would be prohibitive for an embedded system. The system is fully portable and has a simple interface. This new system is also one order of magnitude faster than previous realizations of similar laser scan systems based on event-based sensors.

The implemented algorithm in the FPGA can ask for a new event every six cycles = 120 ns. The processing pipeline can deliver a new distance data each cycle, with a latency of 64 cycles. Since data are being stored in the SRAM and in order to avoid contention, a sequential process of acquiring, computing and storing data has been implemented. This is why the delay has been set to 1.7 μs. The system could work with a temporal resolution of 120 ns, which has been proven to be the minimum sensor event access time (without the illumination value). Sometimes, this time will be greater since some events must be discarded because they are coming from faulty pixels. The processing stage would not be the throughput bottleneck, although as in any pipelined system, it would include some latency. In any case, this latency is several orders of magnitude lower than the latency in a classical full-frame vision system.

Additionally, the SCD approach offers an easy interface, with a processing system that can control the stream data and adjust them to its computation capabilities. The implemented system is also a proof-of-concept of how event-based systems, and particularly selective change-driven vision, can be implemented in real high-speed applications.

Some system limitations must be noted: Object detection with this approach only works when there is movement perpendicular to the sensor plane under the laser line. Furthermore, this first prototype has a relatively low measurement range (distances up to 100 cm) because of the optics and sensor resolution. Additionally, 64 × 64 pixels can be considered a low resolution for some practical applications. Finally, system vibrations and some CMOS sources of noise may affect the distance measurements. 

## Figures and Tables

**Table 1:** Synthesis results.

Parameter	Value
System clock	97.84 MHz
Total logic elements	16,064 (48%)
Total registers	8285 (25%)
Total memory bits	4608 (1%)
Embedded Multiplier 9-bit elements	70 (100%)

## Abbreviations

The following abbreviations are used in this manuscript:

AER	Address Event Representation
CK	Clock sensor signal
CMOS	Complementary Metal Oxide Semiconductor
Comp	Competition sensor signal
ConvNets	Convolutional Neural Networks
DVS	Dynamic Vision Sensor
FPGA	Field Programmable Gate Array
fps	frames per second
FSM	Finite State Machine
Kfps	Kilo frames per second
LiDAR	Light Detection and Ranging or Laser Imaging Detection and Ranging
LPM	Library of Parameterized Modules
LUT	Look-Up Table
PLD	Programmable Logic Device
RMSE	Root Mean Square Error
RTS	Random Telegraph Signal
SCD	Selective Change-Driven
SRAM	Static Random Access Memory
ToF	Time of Flight
VLSI	Very Large Scale of Integration
WTA	Winner-Take-All
